# Supplementary material for: Non-volatile electrically programmable integrated photonics with a 5-bit operation
Source: Nat Commun. 2023 Jun 12;14:3465. doi: 10.1038/s41467-023-39180-3 (PMC10261021; doi:10.1038/s41467-023-39180-3)
Supplement: Supplementary file 1 — Supplementary Information [file 41467_2023_39180_MOESM1_ESM.pdf]

Supplementary information

Non-volatile electrically programmable integrated photonics with a 5-bit operation

Rui Chen[1,*], Zhuoran Fang[1], Christopher Perez[2], Forrest Miller[1], Khushboo Kumari[1], Abhi Saxena[1], Jiajiu Zheng[1], Sarah J. Geiger[3], Kenneth E. Goodson[2], Arka Majumdar[1,4,*]

[1]*Department of Electrical and Computer Engineering, University of Washington, Seattle, WA 98195, USA*

[2]*Department of Mechanical and Aerospace Engineering, Stanford University, Stanford, CA 94305, United States*

[3]*The Charles Stark Draper Laboratory, Cambridge, MA 02139, USA*

[4]*Department of Physics, University of Washington, Seattle, WA 98195, USA*

**Email: charey@uw.edu and arka@uw.edu*

This supplementary information includes:

Section 1. Sb_2S_3 thin film characterization

Section 2. Lumerical simulation for Sb_2S_3-SOI hybrid phase shifters and directional couplers

Section 3. Local crystal grains – micrograph image

Section 4. Extra measurement cyclability test results

Section 5. Multilevel performance based on Sb_2S_3 partial crystallization

Section 6. Heat transfer simulation

Section 7. Micrograph images for different intermediate levels

Section 8. The Table shows pulse conditions for 5-bit operation

Section 9. Performance retention after 77 days

Section 10. Sb_2S_3 stripe thickness after liftoff depends on the pattern width

Section 11. Single-pulse vs. multi-pulse switching

Section 12. Nucleation-speed limited initial crystallization and successive faster crystallization

Section 13. Long-pulse amorphization – evidence for large volume amorphization and a path to low operation voltage

Section 14. Validation and Sensitivity of thermal properties in TDTR

Section 15. Comparison to other PCM devices and various other tuning methods

Section 16. System scalability analysis: physical scale, optical metrics and thermal crosstalk

Section 1. Sb_2S_3 thin film characterization

We started with characterizing the thin film Sb_2S_3 using ellipsometry, X-Ray diffraction, and Raman spectroscopy (*Supplementary Fig. 1*). A layer of 60-nm Sb_2S_3 was sputtered on a silicon wafer. The as-deposited a-Sb_2S_3 was characterized first and then switched to the crystalline phase by annealing at 325°C for 10 minutes under nitrogen flow.

The Sb_2S_3 was characterized by ellipsometry, and the data is fitted with Cody-Lorentz models with low mean square error (~5). The fitted complex refractive indices in *Supplementary Fig. 1(a)* show a drastic change in the real part n (~0.7 at 1310 nm), while almost zero κ (0.0003 for amorphous and 0.03 for crystalline phases at 1310 nm) across the entire telecommunication O- and C-band. Therefore, switching the Sb_2S_3 produces a phase-only modulation. The micro-structural phase transition after annealing and the stoichiometry were verified under X-ray diffraction (XRD) and Raman spectroscopy[1], as shown in *Supplementary Fig. 1(b)* and *Supplementary Fig. 1(c)*, by the characteristic lattice constants in the XRD and wavenumber shifts in the Raman spectrum. These characteristics show good agreement with existing literature[2].

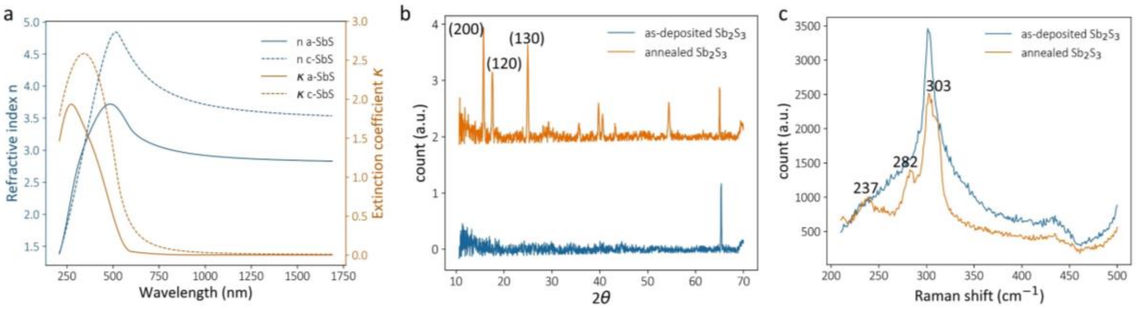

Supplementary Fig. 1: Sb_2S_3 material characterization. (a) Broadband complex refractive index fitted from ellipsometry measurement. (b) X-ray diffraction and (c) Raman spectroscopy measurement for the as-deposited (blue) and annealed (orange) Sb_2S_3 samples. The peak at $2\theta = 65^{\circ}$ for as-deposited Sb_2S_3 comes from the silicon substrate. The characteristic lattice constants and Raman shifts are marked.

Section 2. Lumerical simulation for Sb_2S_3-SOI hybrid phase shifters and directional couplers

S2.1. Sb_2S_3-based silicon phase shifters

Supplementary Fig. 2 shows the simulated π phase shift length L_π and insertion loss for the Sb_2S_3-based phase shifters. The refractive indices of Sb_2S_3 used here were obtained experimentally in the previous section. For a generic 500-nm-wide, 220-nm-high SOI waveguide with 20-nm-thick, 450-nm-wide Sb_2S_3 on the top, an effective index contrast of 0.018 is obtained at 1310 nm in *Figs. S2(a, b)*, indicating a π phase shift length $L_\pi \approx 38\ \mu m$. *Figs. S2(c, d)* shows that a wider and thicker Sb_2S_3 film reduces L_π and does not impact the insertion loss much (~0.23 dB/π). We adopted a generic waveguide width of 500 nm to avoid any extra taper structure, which renders $L_\pi \approx 38\ \mu m$. The calculated mode coupling between the bare silicon waveguide mode and the c-Sb_2S_3-Si hybrid waveguide mode is 99.7% (or -0.013 dB), which is negligible.

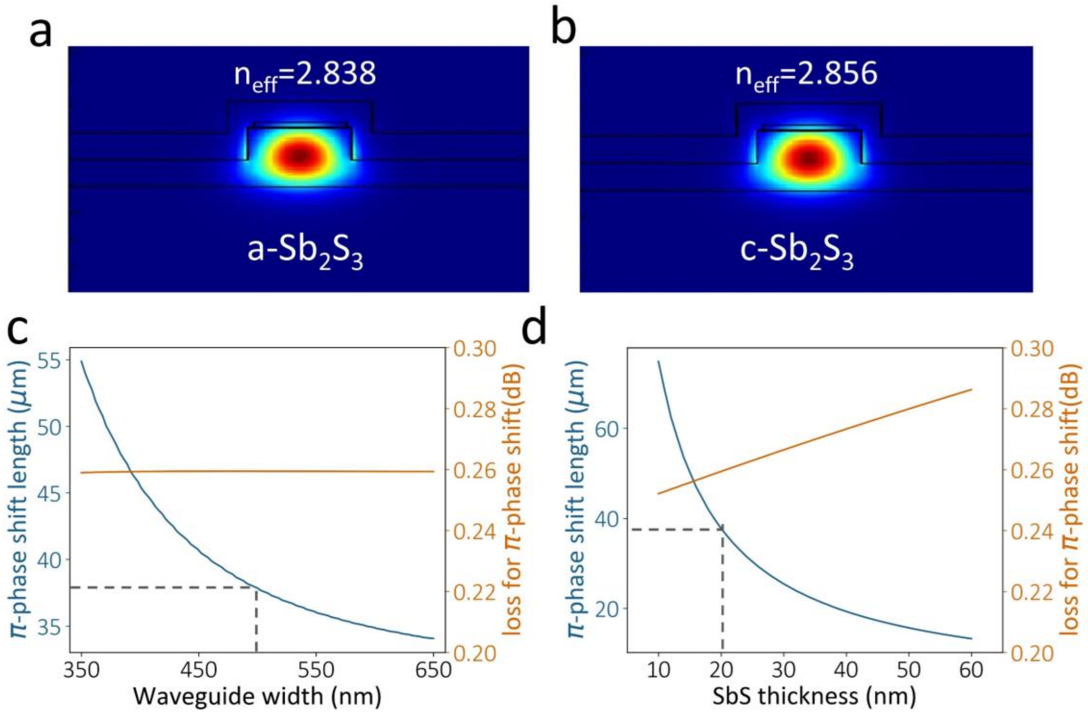

Supplementary Fig. 2: Simulations for Sb_2S_3-on-SOI phase shifters. (a, b) Mode simulations for (a) amorphous and (b) crystalline Sb_2S_3. An effective index contrast of 0.018 is obtained at 1310 nm, indicating a π phase shift length $L_\pi \approx 38\ \mu m$. (c, d) L_π (blue) and excess loss for π phase shift (orange) versus (c) the hybrid waveguide width and (d) Sb_2S_3 thickness. L_π decreases as the increase of both waveguide width and Sb_2S_3 thickness, while the excess loss remains almost invariant. The dashed gray lines represent that we used a waveguide width of 500 nm and an Sb_2S_3 thickness of 20 nm in our experiments.

We note that L_π could be further reduced by exploiting a wider or thicker Sb_2S_3. Although thick Sb_2S_3 gives a more compact device footprint, the complete phase transition is more difficult. The vertical temperature gradient at thick Sb_2S_3 films could lead to Sb_2S_3 ablation at the bottom while not high enough temperature for amorphization at the top. Unintentional re-amorphization could also happen with a thick Sb_2S_3 layers. Moreover, recrystallization could also occur because of the crystallization kinetics and finite heat diffusion time[3]. To accommodate this tradeoff, we pick 20 nm as the experimental thickness, providing both a complete, repeatable Sb_2S_3 phase transition and a relatively compact L_π of 38μm. Since this is the first experiment to switch Sb_2S_3 electrically, we chose a conservative thickness of 20 nm. Considering the good switching behavior observed in our experiment, a thicker Sb_2S_3 could be used in future experiments to provide even more compact devices. The largest thickness of Sb_2S_3 films that could be switched completely require more experimental exploration.

S2.2. Sb_2S_3-based tunable asymmetric directional coupler

The asymmetric directional coupler is designed using the Lumerical Mode simulator and the coupled-mode theory. The idea has been depicted in the main text and the literature[4–6]. *Figs. S3 (a, b)* show two main optimization steps: (i). find the Sb_2S_3-loaded waveguide width with a fixed Sb_2S_3 thickness so that it is phase matched with a bare SOI waveguide; (ii). obtain a high transmission at the bar port when switching the Sb_2S_3 by optimizing the gap between two waveguides to allow the coupling length in the bar state to be an even multiple of the coupling length in the cross state[4]. Unlike the 1 × 2 coupler in ref[4], here we consider a 2 × 2 directional coupler, where light comes in from both input ports. To achieve an input port independent performance, the Sb_2S_3-SOI hybrid waveguide and the bare SOI waveguide are phase matched when Sb_2S_3 is in the crystalline phase. Intuitively, the light interacts with the slight loss of the Sb_2S_3 waveguide half of the L_c regardless of the input port in this configuration.

After accomplishing the optimization in the Lumerical MODE simulator, we ran a Finite Difference Time Domain (FDTD) simulation to verify the design, and the results are in *Figs. S3 (c-f)*. The transmission spectra in *Figs. S3 (c, e)* show that a bar (cross) state is achieved by controlling the phase of Sb_2S_3 to amorphous (crystalline). At 1310 nm, the insertion loss is around 0.1 dB and 0.3 dB for the bar and cross state, respectively, and the extinction ratio is larger than 15 dB in both states. The higher insertion loss for the cross-state is mainly due to the slight material absorption of c-Sb_2S_3. The inset in *Supplementary Fig. 3 (e)* shows that our device performance is symmetric and independent of the input port. *Figs. S3 (d, f)* shows the

field propagation profile corresponding to *Figs. S3 (c, e)*. Again, we can verify an input-port independent performance.

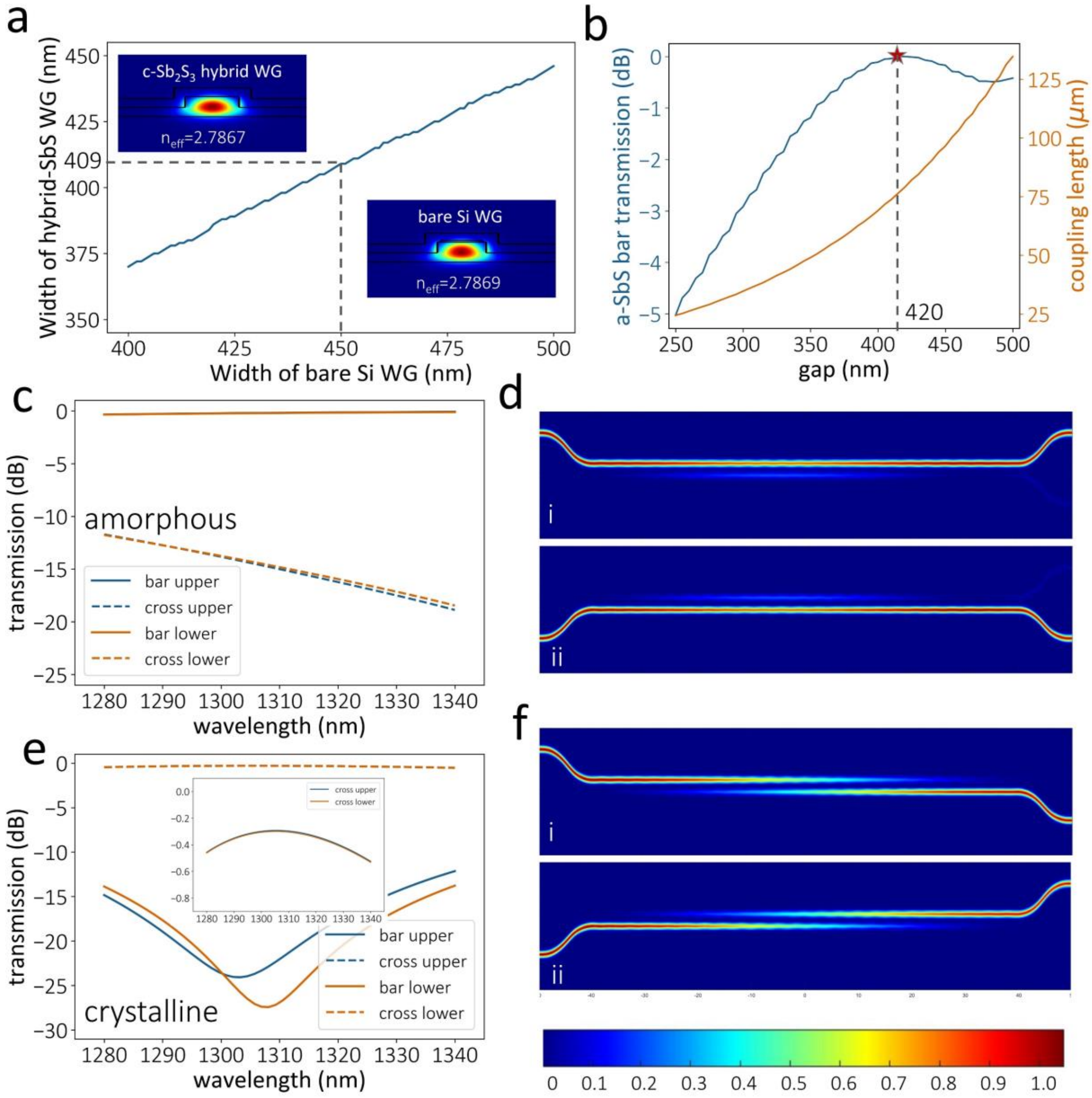

Supplementary Fig. 3: Simulations for Sb_2S_3-on-SOI 2 × 2 tunable directional couplers with low insertion loss and large extinction ratios. (a) Phase-matching widths for c-Sb_2S_3-SOI and bare SOI waveguide. The dashed gray line shows that a 450-nm-wide bare SOI waveguide is phase matched with a 409-nm-wide c-Sb_2S_3-SOI waveguide. Their mode profiles are shown in the insets with a similar effective index, indicating good phase matching. (b) The gap (blue) is optimized to maximize bar port transmission when the Sb_2S_3 is switched into its amorphous phase. The coupling length L_c for the cross state (orange) is also obtained based on the MODE simulation and coupled-mode theory. An optimal gap of 420 nm is picked, corresponding to $L_c \approx 79\mu m$. (c - f) FDTD simulation results. (c, e) Transmission spectrum and (d, f) field profile for (c, d) amorphous- and (e, f) crystalline-Sb_2S_3. (i) and (ii) show the field profile when inputting light from the upper and lower port, respectively. The inset in (e) shows an input-port independent performance for the cross-state.

On the contrary, if the waveguides are phase matched for a-Sb_2S_3, the loss would be much higher when light inputs from the Sb_2S_3 waveguide. *Supplementary Fig. 4* shows the simulated spectrum of an optimized design, where 20-nm a-Sb_2S_3 is phase-matched with a bare SOI waveguide. As shown in the zoomed-in bar transmission plot *Supplementary Fig. 4 (b)*, a difference of 0.3 dB between two input ports is observed when Sb_2S_3 is in its crystalline phase. This seemingly slight asymmetry could lead to unintentionally unbalanced optical paths in a large-scale PIC system.

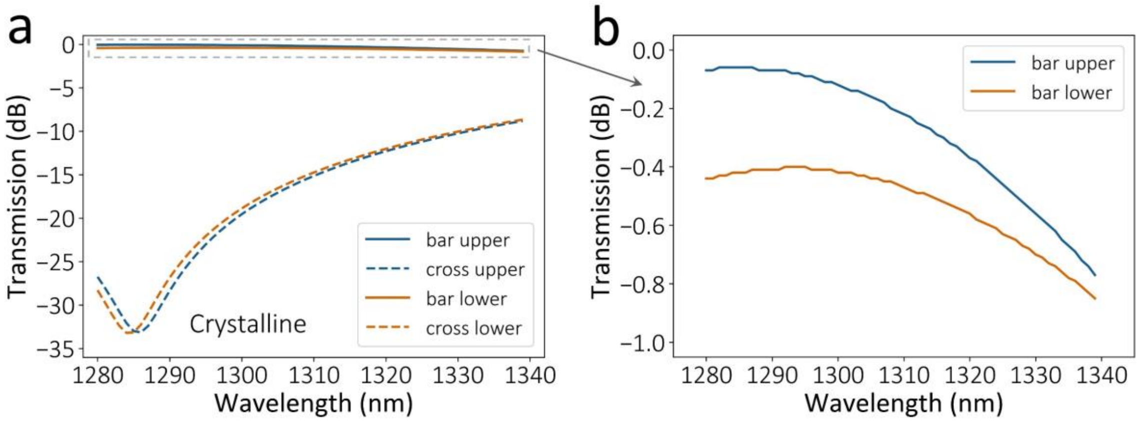

Supplementary Fig. 4: Phase matching a-Sb_2S_3-SOI waveguide with bare SOI waveguide results in an asymmetric (input port dependent) bar state when switching the Sb_2S_3 to its crystalline phase. (a) Transmission spectrum for c-Sb_2S_3. (b) Zoomed in transmission spectrum at the bar port shows a transmission difference of 0.3dB between two input ports.

We adopted the relatively thin Sb_2S_3 with 20 nm thickness to ensure a complete phase transition. However, such a thin PCM layer provides limited tunability, resulting in a large device footprint. Fortunately, we note the slow crystallization speed of Sb_2S_3 could potentially enable a much thicker PCM layer to crystallize (see Supplementary Section 2.1), which will shrink the device size. Using the same design methodology, we numerically designed asymmetric directional couplers with Sb_2S_3 thickness ranging from 30 nm to 50 nm in *Supplementary Table 1*. Remarkably, the designed directional coupler with 50-nm-thick Sb_2S_3 shows a significantly reduced coupling length (~34μm).

Supplementary Table 1: Performances of Sb_2S_3 directional couplers using different Sb_2S_3 thicknesses

t_{SbS} *(nm)*	W_b *(nm)*	W_h *(nm)*	*gap (nm)*	L_c *(μm)*
20	450	409	420	79
30	450	395	370	55
40	450	383	330	41

50	450	373	305	34

Note: t_{SbS}, W_b, and W_h represent the Sb_2S_3 thickness, and widths of bare SOI and hybrid Sb_2S_3-SOI waveguide, respectively.

S2.3. Estimate loss of asymmetric directional coupler using experimentally extracted Sb_2S_3 loss

We extract the actual loss of c-Sb_2S_3, including the crystal grain scattering loss, by match the loss measured in the high-Q ring resonator (0.76 dB/μm) with MODE simulation. We obtained an extinction coefficient $\kappa' \approx 0.015$, compared to ellipsometry measurement result of $\kappa = 0.005$ at a wavelength of 1310 nm. We then estimate the actual loss of the asymmetric directional coupler using the new complex refractive index. *Supplementary Fig. 5* shows the simulation results, where the insertion loss is around 0.85 dB and the extinction ratio is 25 dB. We note the amorphous Sb_2S_3 loss is very small in the experiment, so the initial simulation result still holds (0.1 dB insertion loss and 15 dB extinction ratio).

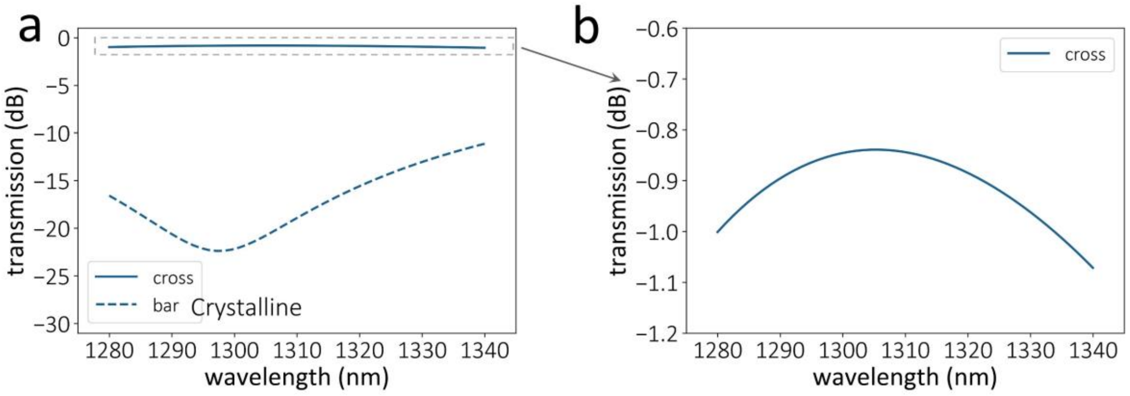

Supplementary Fig. 5: Simulated transmission spectrum for asymmetric directional coupler. (a) Simulation results with the ring resonator measured c-Sb_2S_3 loss, showing high extinction ratio > 20 dB. (b) Zoomed-in plot to show the insertion loss. The insertion loss increases from 0.3 dB to 0.85 dB due to the high material absorption loss used.

S2.4. A multimode interferometer (MMI)

A 120-nm partially etched MMI was designed using Lumerical Eigen-Mode Expansion (EME) Method. The design was further tweaked and verified with 3D FDTD simulations. The optimized multimode device parameters are annotated in *orange in Supplementary Fig. 6 (a). Figs. S6 (a, b)* show the simulated electric field propagation profile and the transmission spectra at both output ports. The optimized MMI exhibits an insertion loss < 0.1 dB at 1310 nm and a

1-dB bandwidth > 60 nm, as shown in *Supplementary Fig. 6 (b)*. The measured transmission spectra are presented in *Supplementary Fig. 6 (c)*. The slight spectral fluctuation (~0.1 dB) is attributed to the interference between back-reflected light at the interface between the single mode tapers and the multimode region.

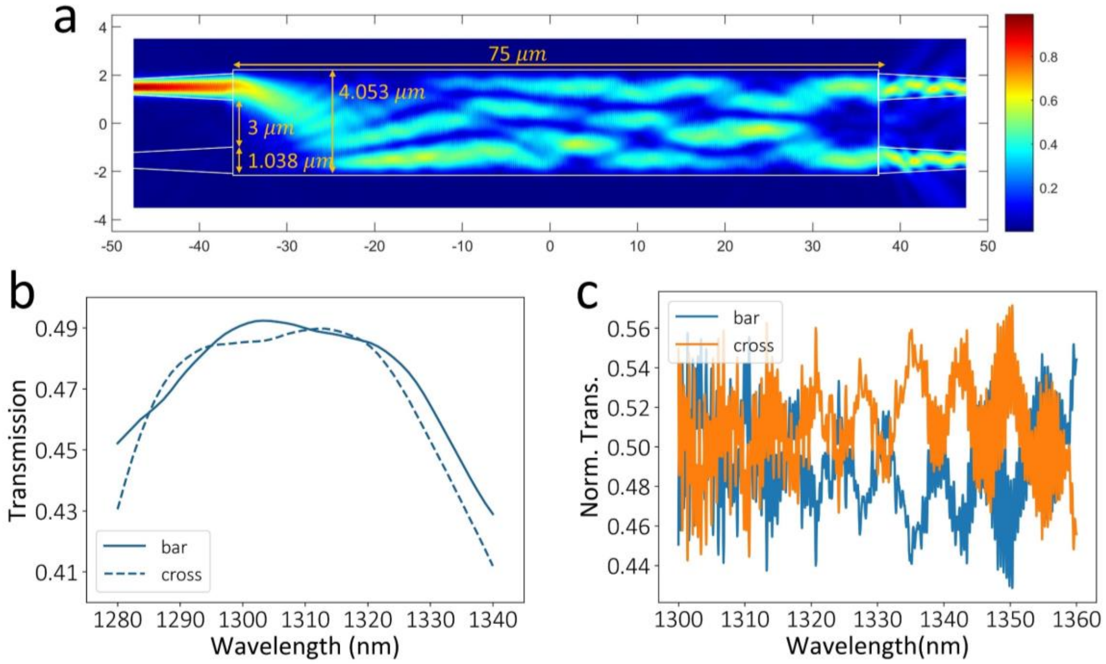

Supplementary Fig. 6: Low-loss 50:50 multimode interferometer (MMI) at 1310 nm. (a) Field profile and (b) transmission spectrum using FDTD simulation. The white dotted line outlines the device shape. (c) Measured transmission spectrum of the fabricated device.

Section 3. Local crystal grains – micrograph image

In *Supplementary Fig. 7*, we took a micrograph image of 60-nm-thick Sb_2S_3 on a blanket silicon chip, where the Sb_2S_3 films were annealed under 325°C and in the crystalline phase. The micrograph image shows a non-uniform surface with a lot of local crystal grains and even flower-shaped patterns (circled in red), which could lead to unintended scattering loss for in-plane light propagation. We hypothesize that the crystal grains form because crystalline Sb_2S_3 has a density reduction of around 24% compared to its amorphous form[7]. Hence, a significant strain is induced during the phase transition, and results in a non-uniform surface. We also speculate that the flower-like patterns occurs near the nuclei.

It is unclear what the c-Sb_2S_3 surface looks like on our devices because those 20-nm 450-nm-wide Sb_2S_3 films are too small to image optically. The 40-nm-thick encapsulation alumina further prevents the accurate usage of SEM. Further material study is required to quantitatively understand the crystalline patterns formed.

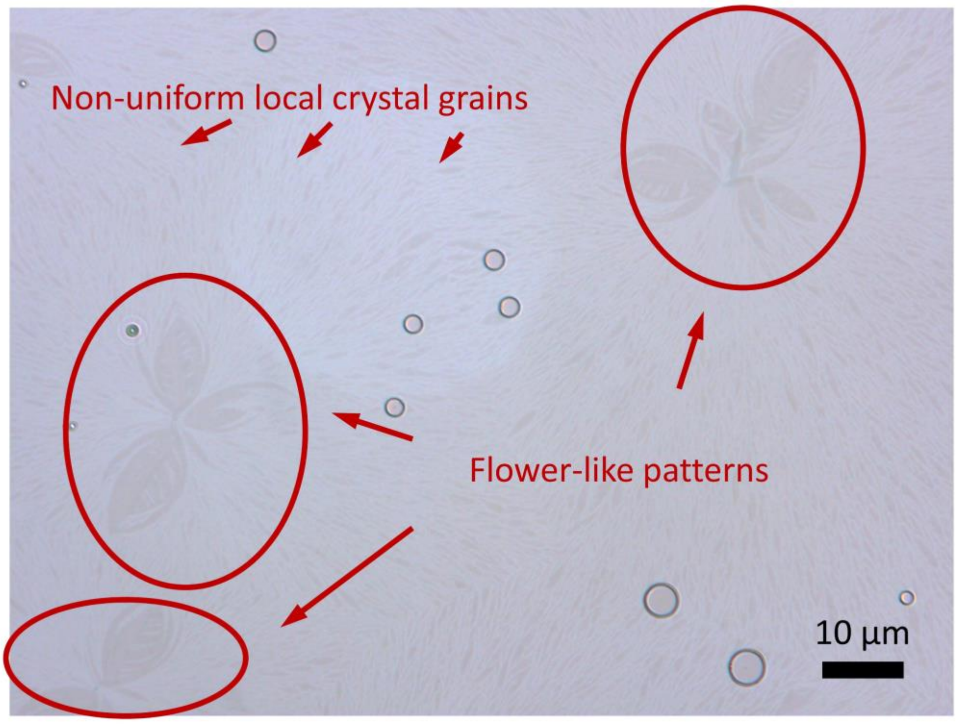

Supplementary Fig. 7: Non-uniform surface forms after the annealing due to the density difference between amorphous and crystalline Sb_2S_3. Some flower-shaped patterns are observed. We speculate the nonuniform surface is due to the large density difference between a- and c-Sb_2S_3, and the flower-shaped patterns occur near the nuclei.

Section 4. Extra device measurement results

MZI current-voltage characteristics and cyclability result – 100 cycles

We measure the current-voltage (IV) response on an MZI arm with a doped region length of 30 μm by applying a voltage from -0.5 to 1.0 Volt. The measured IV-curve shows a typical rectified shape and has a turn-on voltage of around 0.8 V and resistance of 50 Ω. The Sb_2S_3 phase shifter in an MZI was switched reversibly by alternatively applying SET and RESET pulses. We note this cyclability test was performed with a single pulse scheme, so a significant variation was observed compared to *Fig. 4* in the main text, where three pulses were used for each amorphization or crystallization. Yet, we found 100 consecutive switching events (*Supplementary Fig. 8*) where the device was switched with a high extinction ratio of ~15 dB.

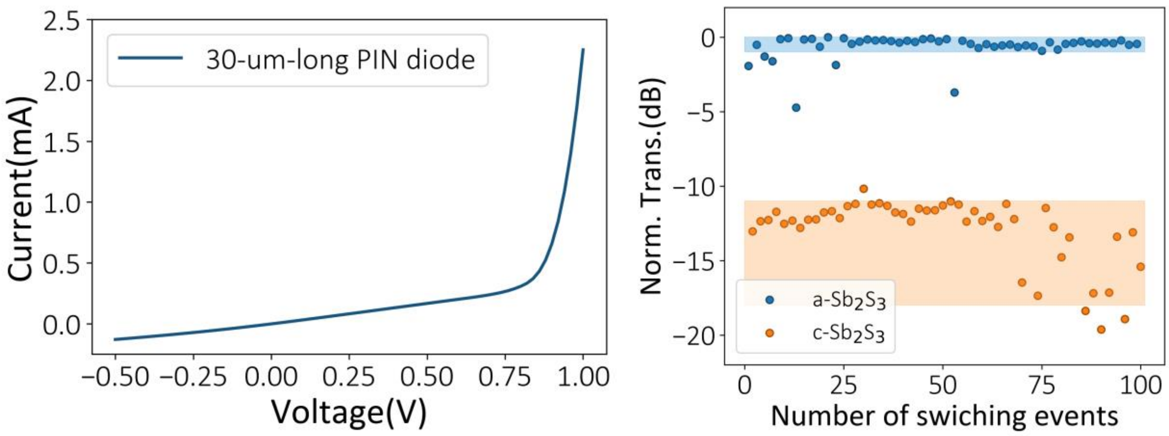

Supplementary Fig. 8: Extra measurement results on MZIs. (Left) I-V characteristics of the 30-μm-long PIN diode, showing a turn on voltage of 0.8 V and a resistance of 50 Ω. (Right) The Sb_2S_3-based phase shifter in an MZI is experimentally switched between amorphous (blue) and crystalline (orange) phases for 100 switching events. Each switching event was triggered with a single pulse.

Asymmetric directional coupler transmission before and after 2,300 switching events

We switched the asymmetric directional coupler for 2,300 switching events. However, some drift in the optical measurement setup occurred, so only 1,600 events were reported in the main text. In this section, we compare the SEM image and the transmission spectrum before and after 2,300 switching events, and give an explanation of the performance degradation or the transmission drifting.

Figs. S9 (a, b) show the scanning electron-beam micrograph images of Sb_2S_3 directional couplers before and after the cyclability test. We note that the black dots on the lower waveguide (*Supplementary Fig. 9 (a))* is likely the crystalline Sb_2S_3 nuclei formed during the rapid thermal annealing. The rough Sb_2S_3 edge in *Supplementary Fig. 9 (b)* indicates that switching the relatively large volume of the Sb_2S_3 stripe many times results in a distorted Sb_2S_3. This is

because the thin film always tends to minimize the surface energy during melt-quench process, hence leads to sinusoidal edge. This phenomena is known as Rayleigh instability of fluid motions[8]. The result of such surface-energy-induced instability could be discrete circular patterns, having been used for advanced nano-fabrication[9]. Therefore, to further improve the endurance of our devices, one possible approach is to pattern the long Sb_2S_3 stripes to subwavelength gratings to ensure a lower initial surface energy and a low excess loss. We note that this subwavelength grating idea has been experimentally demonstrated in other works using GST[10–12], hence could be a future direction to improving the presented work.

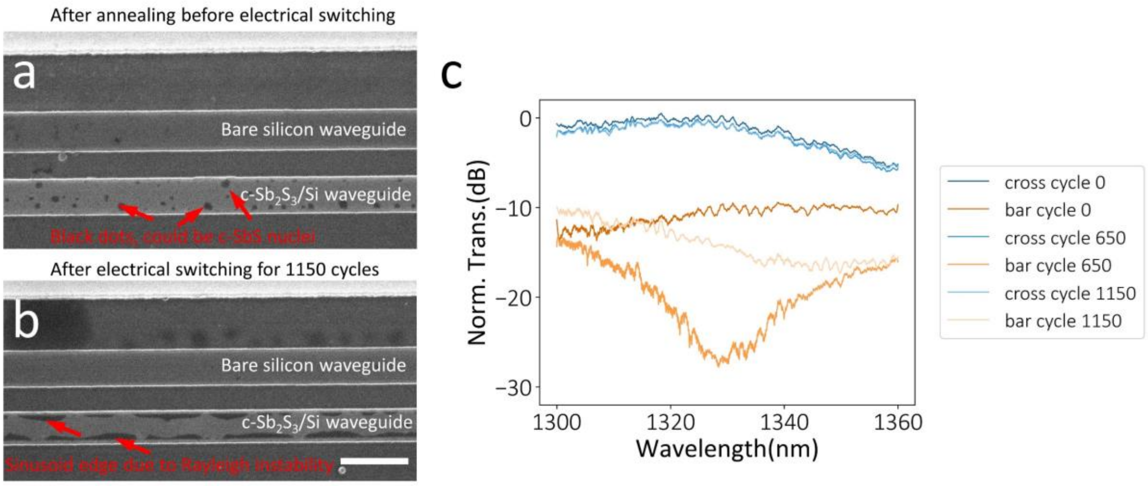

Supplementary Fig. 9: Only slight device degradation occurred after 1,150 cycles (2,300 switching events), and the SEM images suggest it is due to thermal reflowing. (a, b) SEM images of a device after (a) a single rapid thermal annealing and (b) 1,150 cycles. Scale bar: 1 µm. (c) Transmission spectra before, in the middle, and at the end of the cyclability test. No significant performance degradation in the cross port (blue) was observed after switching the asymmetric directional coupler for more than 1,150 cycles. The variation in the bar port could be attributed to the change in Sb_2S_3 distribution due to the thermal reflowing or Rayleigh instability issue.

The transmission spectra before and after the cyclability test are shown in *Supplementary Fig. 9 (c)*. A minor drift in the spectra could be attributed to the thermal reflowing, which changes the Sb_2S_3 shape, hence breaking the phase matching condition. Nevertheless, the device still shows a low insertion loss (< 1dB degradation) and high extinction ratio (~ 15 dB).

Section 5. Multilevel performance based on Sb_2S_3 partial crystallization

By sending in relatively low voltage crystallization pulses (voltage 3.1 V, duration 200 ms, leading-edge 10 ms, falling edge 100 ms), we obtained partial crystallization performance in *Supplementary Fig. 10*. Such behavior could be explained by the growth-dominant nature of Sb_2S_3[4]. The growth rate could be controlled by the temperature (pulse voltages); the total crystallization volume could be controlled with the duration of crystallization pulses. Further engineering the pulse parameters could potentially achieve more operation levels.

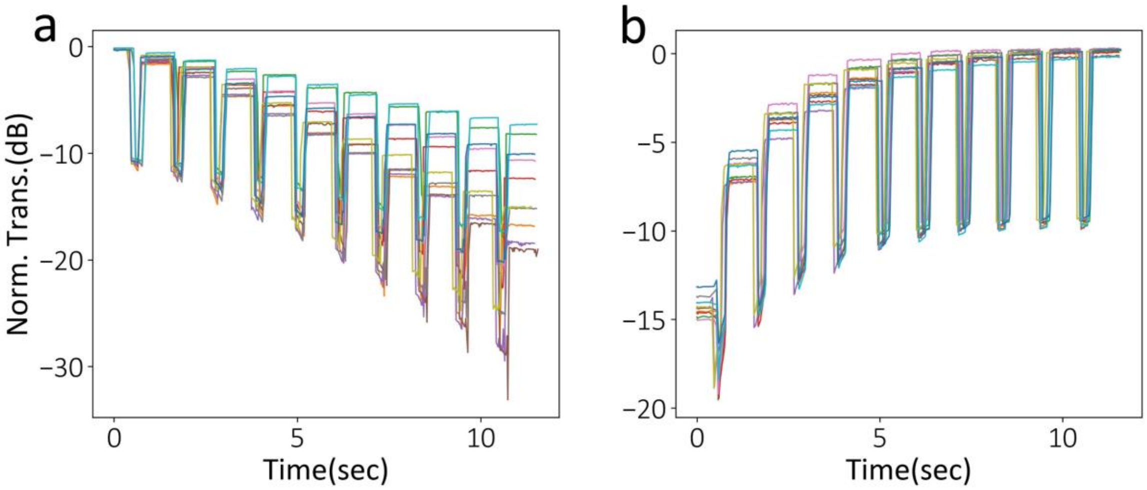

Supplementary Fig. 10: Stepwise partial crystallization using identical, low amplitude, and long duration pulses with a one-second interval. Continuous time trace measurement at (a) bar and (b) cross port of an asymmetric directional coupler. The experiment was repeated for 10 times at each output port, represented by different colors, and the multilevel characteristic occurred in all experiments.

Section 6. Heat transfer simulation results

A heat transfer simulation in COMSOL Multiphysics is presented in this section[6,13–16]. The large thermal gradient between Sb_2S_3 and its surroundings leads to a fast cooling rate. Such a fast cooling rate ($> 10^9\ K/s$ [17]) is necessary for complete amorphization to take place. Simulation shows that the temperature returns to near room temperature within a few μs (*Supplementary Fig. 11*). The μs-level thermal relaxation time rules out any residual heat when we applied the one-second interval electrical pulses for stepwise multilevel amorphization.

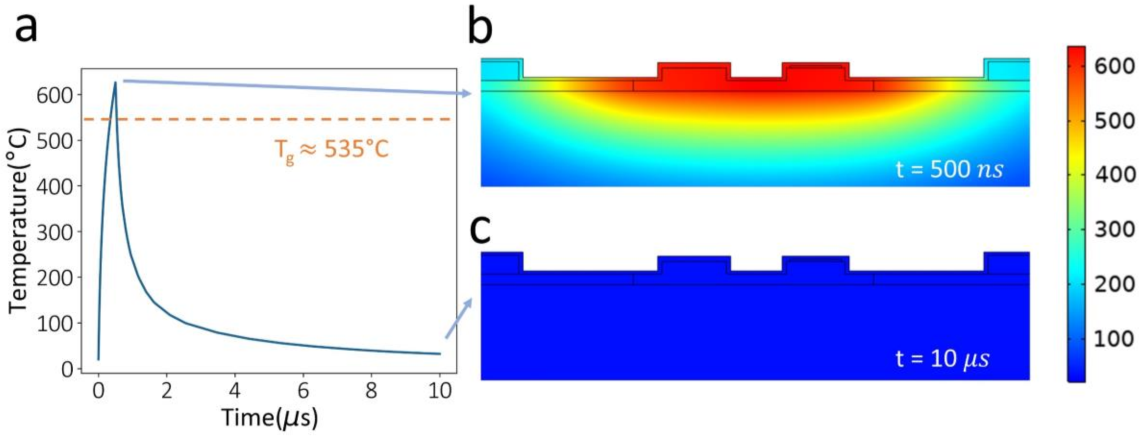

Supplementary Fig. 11: The thermal relaxation time for a 500 ns amorphization pulse is around several μs. (a) Time-dependent heat transfer simulation results at the Sb_2S_3 thin film. (b, c) The thermal profiles at (b) t = 500 ns and (c) t = 10μs.

Section 7. Micrograph images for different intermediate levels

We inspected devices at different intermediate levels under the microscope in *Supplementary Fig. 12*. One can see a gradual increase of amorphous Sb_2S_3 as the degree of amorphization increases. The amorphous Sb_2S_3 is not located precisely in the center of the long strip, where the temperature is the highest. We attribute this to the possible non-uniform doping profile and recrystallization of Sb_2S_3 near the center.

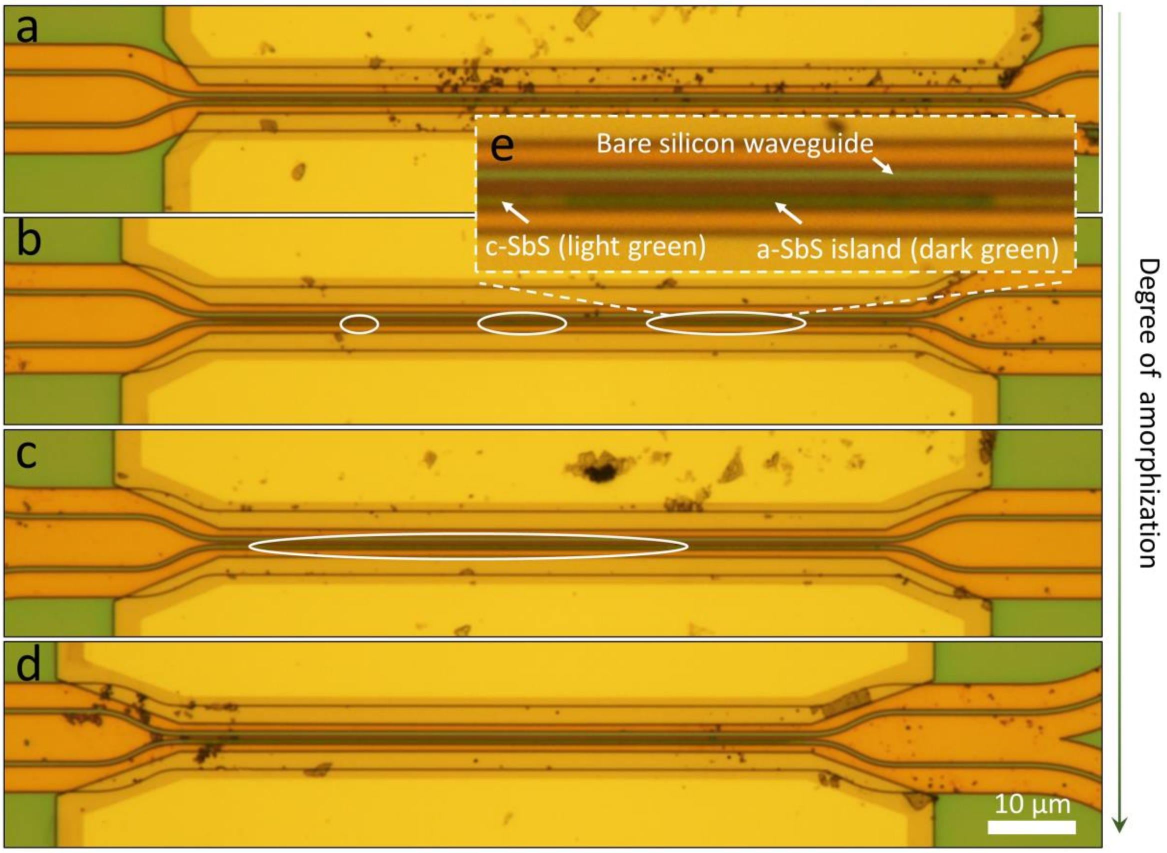

Supplementary Fig. 12: Micrograph images for directional couplers in different intermediate levels. The levels are set using identical pulses with one-second intervals. The degree of amorphization increases from (a) to (d). The dark (light) green regions on the lower waveguide are a(c)-Sb_2S_3. Islands of a-Sb_2S_3 (circled in red) are visible in (b) and (c) and continue to grow. (e) A zoom-in picture of the right-most circled region in (b). A clear boundary between a- and c-Sb_2S_3 is observed in the Sb_2S_3/Si hybrid waveguide (the lower waveguide).

Section 8. Table shows pulse conditions for 5-bit operation

We engineered the pulse condition dynamically to achieve the high 0.5-dB precision. We started with low voltage (9.65 V) that could barely trigger the amorphization and gradually increase the amplitude. In the meantime, we monitored the transmission after sending each pulse. If the transmission is not changed, we increase the voltage slightly (by 0.05 V). If the transmission changes, but smaller than (0.5 ± 0.1) V (this value is our target), we continue to send more pulses until the targetted transmission is obtained. If the transmission is changed by larger than (0.5 + 0.1) V, we partially crystallize the device and repeat the previous steps. We note that it is necessary to monitor the transmission and dynamically change the pulse conditions to mitigate the stochastic nature of PCMs[18]. The following *Supplementary Table 2* shows the pulse conditions in the 5-bit operation experiments.

Supplementary Table 2: **Pulse conditions for 5-bit operation in five experiments (Unit: Volts).** Pulses all have a duration of 550 ns, but the amplitude is increasing gradually. Either single or multiple pulses were applied for each level.

**Level**	**Exp 1**	**Exp 2**	**Exp 3**	**Exp 4**	**Exp 5**
**1**	9.7	9.65	9.75	9.6	9.6
**2**	9.7, 9.8	9.8	9.75 × 3	9.6	9.62, 9.63, 9.64
**3**	9.8	9.8	9.8	9.6 × 2	9.65
**4**	9.75	9.75	9.8	9.65	9.65, 9.62
**5**	9.75	9.75	9.8	9.65	9.62
**6**	9.75 × 2	9.75	9.8	9.65	9.65
**7**	9.7, 9.75	9.75 × 2	9.8	9.65, 9.6	9.65
**8**	9.75, 9.72	9.75	9.8	9.7	9.65
**9**	9.72 × 4	9.75	9.75	9.7	9.65, 9.63
**10**	9.72 × 3	9.75	9.75 × 2	9.67	9.67 × 2
**11**	9.75, 9.73 × 2	9.75	9.75	9.67	9.69
**12**	9.73× 3	9.75	9.75 × 2	9.67	9.7, 9.67
**13**	9.73 × 3	9.75 × 2	9.8	9.67	9.72
**14**	9.73 × 3	9.75	9.75	9.68, 9.66	9.72 × 2
**15**	9.73 × 3	9.75 × 2	9.8	9.68, 9.67	9.72
**16**	9.74 × 3	9.75 × 2	9.75	9.69	9.72
**17**	9.76, 9.8	9.75 × 2	9.8	9.69,9.7	9.72
**18**	9.8 × 2	9.8	9.8, 9.75	9.7,9.67 × 2	9.72, 9.7
**19**	9.8 × 2	9.8	9.75	9.7	9.7
**20**	9.8 × 3	9.8	9.8	9.7	9.72
**21**	9.8 × 2	9.8	9.8, 9.75	9.7	9.73

**22**	9.8 × 2	9.8 × 2	9.8	9.7 × 3	9.75 ×2
**23**	9.8 × 2	9.8	9.8	9.73	9.75 × 2
**24**	9.85	9.8 × 2	9.8	9.73	9.78
**25**	9.85	9.85	9.85	9.75	9.8 × 2
**26**	9.85	9.85	9.8	9.75	9.8
**27**	9.85	9.85	9.85	9.75	9.82
**28**	9.85	9.85	9.85	9.75 × 2	9.85
**29**	9.85	9.85	9.85	9.77	9.8
**30**	9.85	9.85	9.8	9.77 × 3	9.85
**31**	9.85	9.85	9.85	9.77 × 3	9.85
**32**	9.85	9.85	9.85 × 2	9.77 × 3	9.85

Section 9. Performance retention after 77 days

Our device is non-volatile under the ambient environment. We measured a device after leaving it in the ambient (open in the air) for 77 days and compared its performance in *Supplementary Fig. 13*. The device retains its performance excellently, with slight variation, which the small difference in the grating coupler coupling condition (coupling angle) might cause.

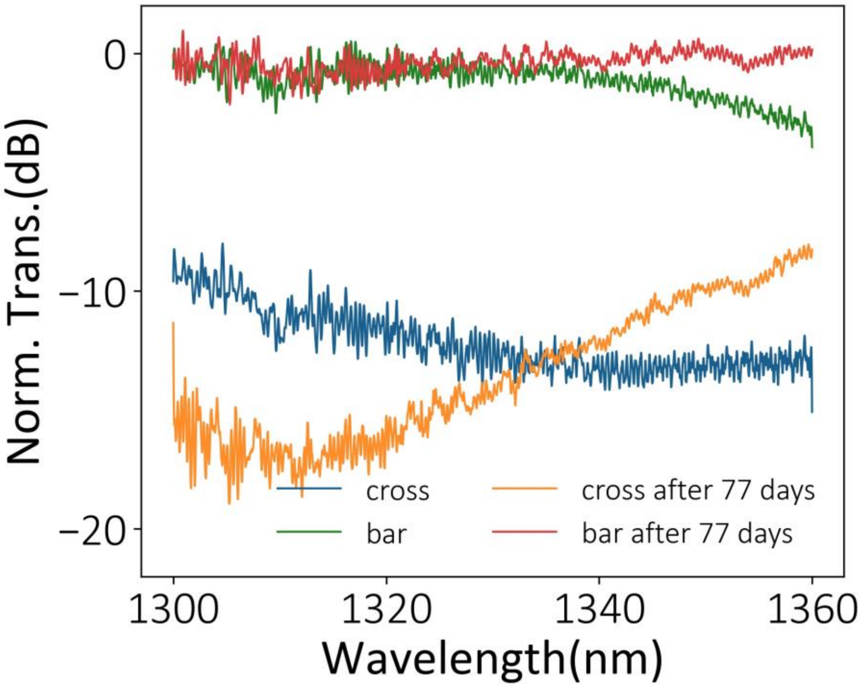

Supplementary Fig. 13: An asymmetric directional coupler's characteristics was retained over 77 days.

Section 10. Sb_2S_3 stripe thickness after liftoff depends on the pattern width

We find that a narrow resist trench for Sb_2S_3 deposition and liftoff leads to a reduced Sb_2S_3 thickness than blanket Sb_2S_3 deposition. This could be attributed to that part of Sb_2S_3 was blocked by the resist due to a deep trench. We fabricated a chip with different patch width (300, 400, 500 nm) and measure the thickness after Sb_2S_3 liftoff. The measured results (*Supplementary Table 3*) shows a reduction factor of around 0.5 compare to blanket deposition. We also measured the widths of the Sb_2S_3 widths which agree reasonably well with the Ebeam defined widths. Since this is not the main interest of this measurement, we only measured part of the Sb_2S_3 stripes, and the ones not measured are denoted as '-'.

Supplementary Table 3: Sb_2S_3 thickness reduces after liftoff measured by AFM (Unit: nm)

Deposited width	Width after Liftoff	Deposited thickness	Thickness after liftoff
**300**	373	30	13.8
**300**	354	30	14
**400**	471	30	16.9
**400**	471	30	17
**300**	-	40	16.4
**400**	413	40	19.5
**300**	-	50	21
**400**	-	50	26
**400**	471	50	25.9

Section 11. Single-pulse vs. multi-pulse switching

In our experiment, single pulses could not trigger a reliable, complete phase transition. We sent in a single pulse for SET/RESET. The final state shows a significant variation (*Supplementary Fig. 14 (a)*). This could be attributed to the multiple crystalline phases and incomplete thermal process due to the relatively thick Sb_2S_3. We tackled this issue by sending two or three pulses to trigger a complete phase transition. The resulting transmission spectra of another ring resonator are shown in *Supplementary Fig. 14 (b)*. This multi-pulse switching scheme achieved a much more consistent performance across ten cycles. We emphasize that this behavior is distinctly different compared to GST or Sb_2Se_3, where we were able to actuate the phase transition under a single shot, in a very similar PIC. While we provided a hypothesis for such behavior, more material studies are warranted to explain the behavior.

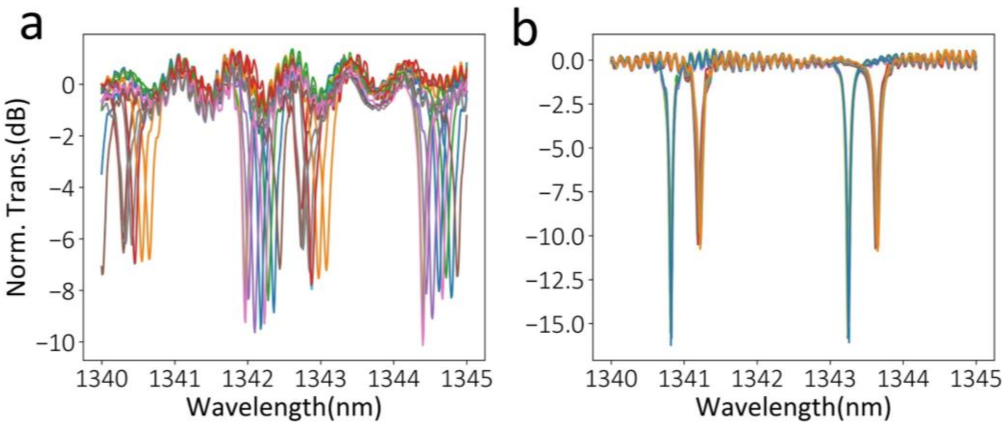

Supplementary Fig. 14: Single SET (RESET) pulses could not reliably trigger complete crystallization or amorphization. (a, b) Transmission spectra of a ring resonator switched with (a) single SET(RESET) pulses and (b) three identical SET(RESET) pulses with one-second intervals. Different colors represent different experiments.

Section 12. Nucleation-speed limited initial crystallization and successive faster crystallization

Experimentally, we observed a very slow Sb_2S_3 crystallization for the first time, where even 500 μs pulses could not trigger the crystallization. The devices were then crystallized with DC voltage or pulses with very long durations such as 200 ms. This could be explained by the small enthalpy of fusion of Sb_2S_3 (~40.64 kJ/mol[19], compared to fusion enthalpy[20] of GST 625 J/cm^3 or $\frac{625}{\rho}M = \frac{625\ J/cm^3}{5.87\ g/cm^3} \cdot 1026.8\ g/mol = 109.3\ kJ/mol$, where ρ is the density[21] and M is the molar mass of GST), which necessitates a large critical nuclei size to overcome the crystallization energy barrier.

After the first crystallization, however, the successive crystal growth process happens without any requirement for overcoming the energy barrier. We were able to see partial crystallization using much shorter 100-μs pulses, as shown in *Supplementary Fig. 15*. We note this slow crystallization guarantees larger volumes of Sb_2S_3 amorphized again without recrystallization and is thus could be beneficial for photonic applications. In the next section, we experimentally show that indeed the amorphization could be triggered with very long pulses.

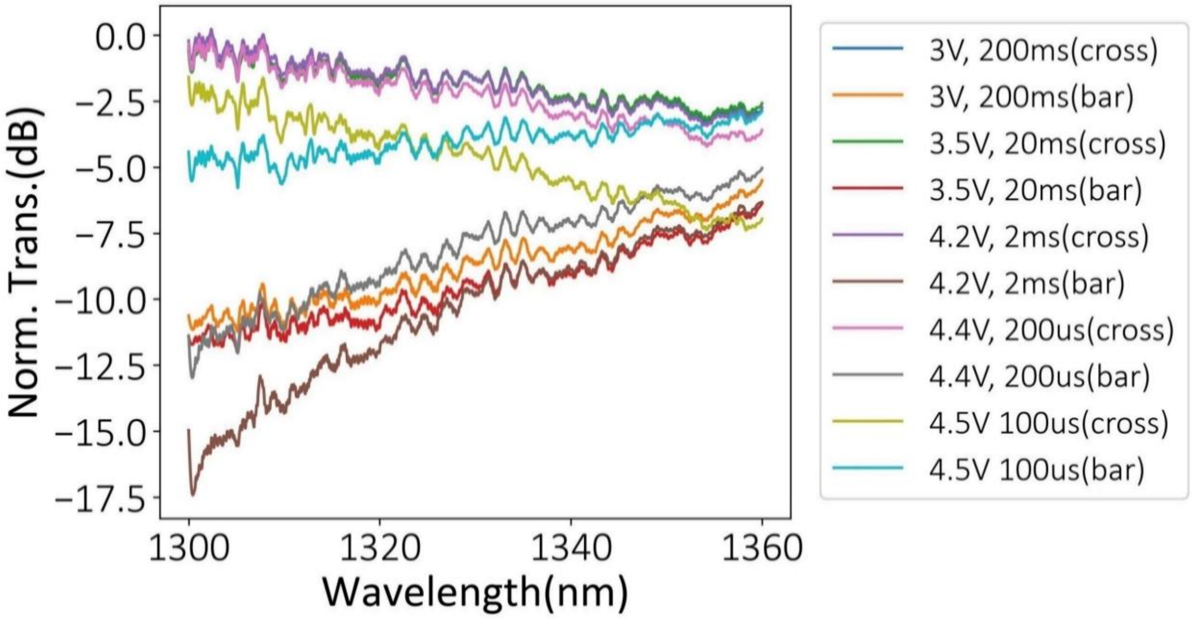

Supplementary Fig. 15: The slow crystallization nature of Sb_2S_3 limits the pulse duration longer than 100 μs. The 100-μs-long pulses were not able to trigger complete crystallization even after the first time. We note again that during the first few cycles of crystallization, only pulses with even much longer duration, such as 200 ms, could trigger the crystallization. This indicates the difficulty in forming initial Sb_2S_3 nuclei and the substantially faster rate of crystal growth compared with nucleation.

Section 13. Long-pulse amorphization – evidence for large volume amorphization and a path to low operation voltage

One appealing aspect of using "slow" PCMs, such as Sb_2S_3, is that the slow crystallization process could avoid any unintentional recrystallization during the melt-quench process, hence could ensure the amorphization for a large volume of PCM. This capability of completely switching large volumes of PCMs is crucial in photonics, where the volume (typically orders of μm^3) are much larger than in electronic applications (typically orders of $(10\ nm)^3$) and takes precedence over the phase transition speed[22].

Here, we verify Sb_2S_3's resistance to unintentional recrystallization by comparing the longest amorphization time of an asymmetric directional coupler to other reported PCM devices. *Supplementary Fig. 16* shows that the Sb_2S_3 on the directional coupler could be amorphized under different pulse durations, ranging from 500 ns to 10 μs. A large degree of amorphization was observed even when we increased the pulse durations to 10 μs. This 10-μs pulse duration is much longer than in the reported GST[6,14,23] (< 200 ns) or SbSe[24] (< 1 μs, but the thickness is 30 nm) switching experiments. This successful amorphization with long pulses supports that Sb_2S_3 is inherently more suitable for large volume amorphization because recrystallization is surpressed. Moreover, the long amorphization pulses relaxed the requirements of high voltage from ~10 V down to 5V, showing a potential path towards CMOS compatible voltage levels (1~2 V).

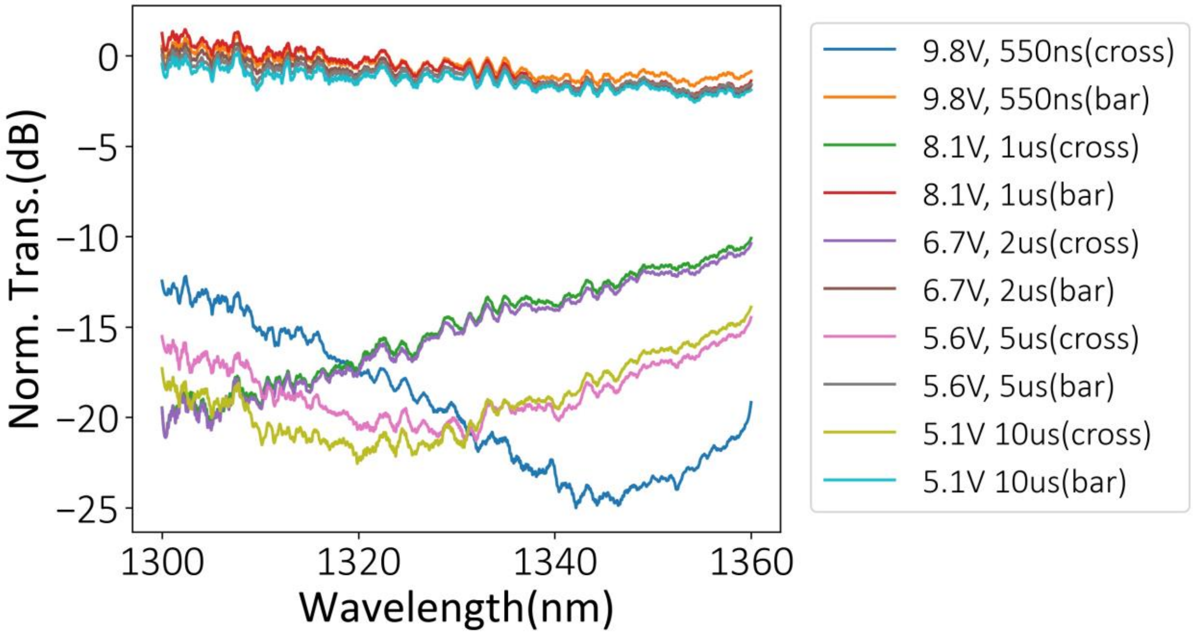

Supplementary Fig. 16: Complete amorphization was triggered with relatively long, 10-μs pulses because of the slow crystallization nature of Sb_2S_3. High-level port (bar port) transmission is consistent

among all the pulse conditions. The low-level port (cross port) transmission variation could be attributed to a tiny portion of the Sb_2S_3 not being switched completely, which could be potentially overcome by optimizing the heater design.

Section 14. Validation and Sensitivity of thermal properties in TDTR

To validate the TDTR system, the thermal conductivity of silicon substrates on which the films were deposited on was determined. With knowledge of the thermal properties of the Pt transducer film itself, found via Van-der Paaw measurements and application of the Wiedemann-Franz Lorrenz Law[25], the thermal conductivity of the silicon substrates was found to be 112.2 $\pm$ 6.8 W m^{-1} K^{-1}, as shown in *Supplementary Fig. 17*.

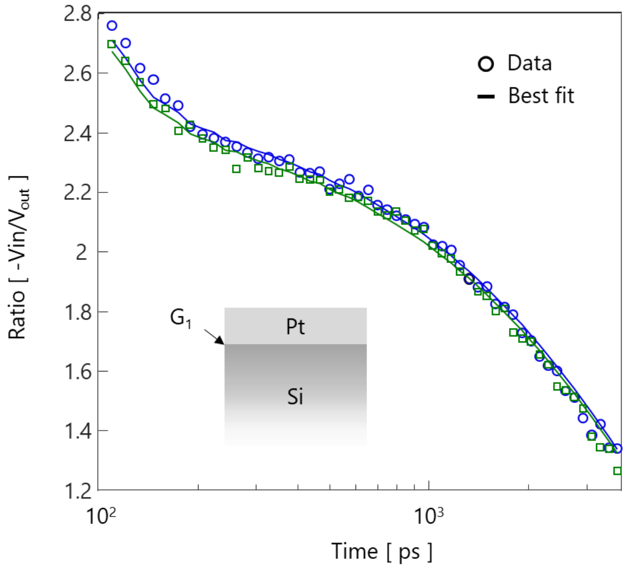

Supplementary Fig. 17: Characteristic model fit for the silicon substrates used in this work. The insets show the thermal model for the samples, with each layer described by its volumetric heat capacity (ρc_p), cross- and in-plane thermal conductivities ($\kappa_\perp/\kappa_\parallel$), layer thickness ($d$), and the boundary conductance to the next layer (G).The Pt-Si G was found to be 128.0 $\pm$ 6.8 *MW m^{-2} K^{-1}*.

The sensitivity to thermal properties in our films was determined following a similar paradigm described in prior works[26]. Specifically, a sensitivity coefficient S is calculated as the logarithmic derivative of the voltage ratio signal ($R = -V_{in}/V_{out}$) from the lock-in amplifier with respect to the parameter of interest x, $S_x = d(ln(R))/d(ln(x))$. Cross-plane thermal transport was found to dominate the thermal signal, and a dual frequency approach for simultaneously extracting thermal conductivity and heat capacity using was implemented to reinforce this point. This measurement involved measuring the thermal response of our films at two different frequencies to note whether we could separate the contributions of thermal conductivity and heat capacity to the overall thermal response[27]. This is because the thermal

conductivity of a material affects the rate at which heat is conducted through the material, while heat capacity affects the amount of heat stored in the material. These two properties have different frequency dependencies, which can allow them to be separated using the dual frequency approach. Here, two or more modulation frequencies are used and either the thermal conductivity or the volumetric heat capacity is iterated, while the other is fit to the 3D heat diffusion model for a multi-layer stack of materials. A crossing point in the data would indicate the properties that satisfy the sensitivities for both parameters. The results of this exercise are shown in *Supplementary Fig. 18*.

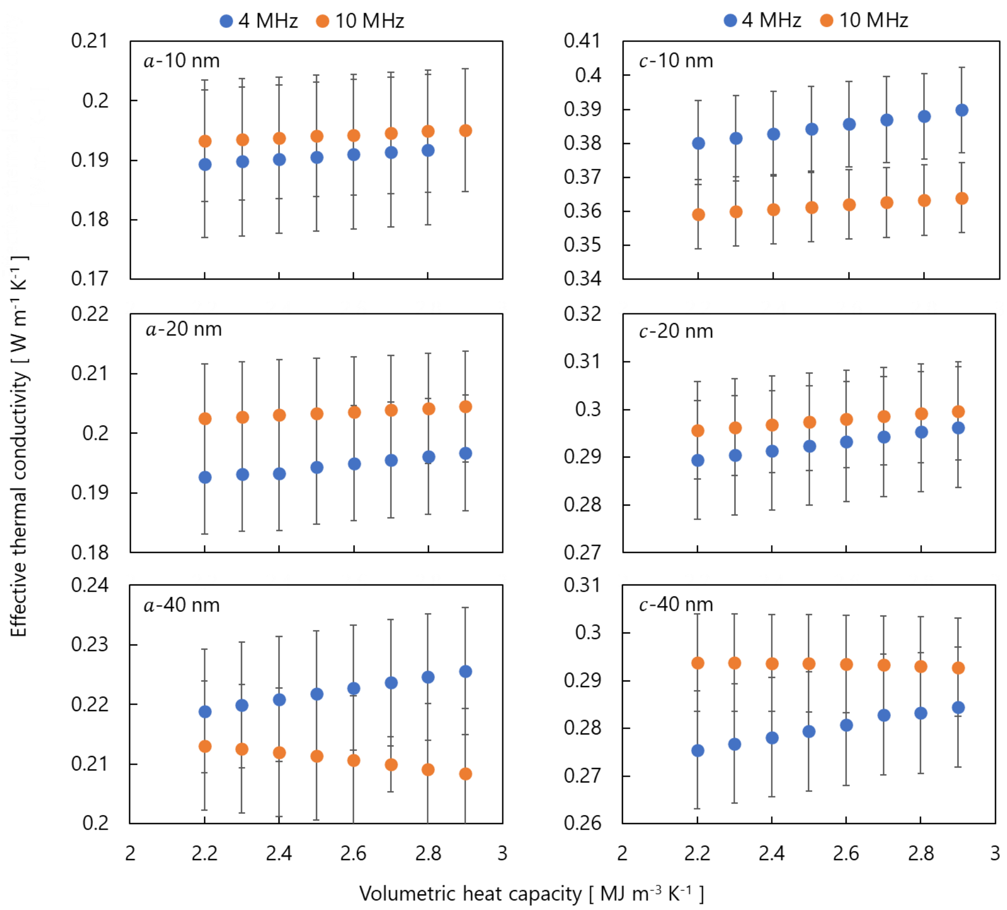

Supplementary Fig. 18: Thermal effective conductivity vs volumetric heat capacity diagrams for the Sb_2S_3 films, with the thicknesses and amorphous/crystallin nature shown in the insets. Multiple pairs of thermal conductivity and volumetric heat capacity are fit using the measured signal with two modulation frequencies (4 MHz and 10 MHz), showing the lack of a crossing point that would indicate sensitivity to both these properties.

As seen in Supplementary Fig. 18, there is no crossing point for the 4 MHz and 10 MHz modulation frequencies, and thus, no simultaneous sensitivity to both properties. On the other hand, we observe that a ~40% change in the volumetric heat capacity result in a ~10% change

in the effective thermal conductivity, thus reinforcing the cross-plane domination of the thermal signal.

As explained in the main text, the intrinsic thermal conductivity was found by measuring the effective thermal resistance of the films as a function of thickness. A linear regression was then used to determine the limit of zero thickness, thus providing the thermal resistance of the interfaces surrounding the Sb_2S_3 films. This thermal resistance can then be subtracted from the total and the intrinsic thermal conductivity can be determined using the expression $d/\kappa_{\perp}$, where d is the film thickness. The results of this analysis are shown in Supplementary Fig. 19, with the combined thermal resistances of the Pt-Sb_2S_3 and Sb_2S_3-Si interfaces shown in the Supplementary Fig. 19(b).

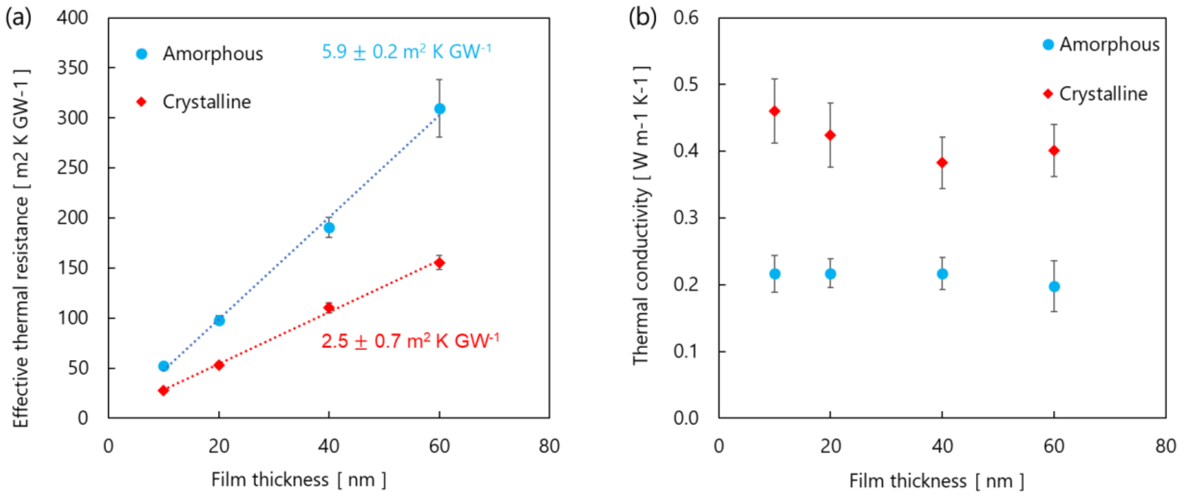

Supplementary Fig. 19: (a) The effective thermal resistance plotted against the film thickness, showing the linear regression used to determine the total thermal boundary resistance. The thermal boundary resistance values for both the amorphous and crystalline are shown in the inset. (b) The intrinsic thermal conductivity upon subtraction of the thermal boundary resistance.

Section 15. Comparison to other PCM devices and various other tuning methods

Supplementary Table 4 compares our device with other PCM devices. We note that only reversibly switchable PIC devices are compared here. We refer the readers to some recent review articles for PCM metasurface works[28,29]. *Table. S4* clearly shows that electrical control is growing rapidly since 2017 and getting more interest in recent years. In addition, while GST is the most commonly used PCM so far, recent works are using wide-bandgap PCMs to eliminate the material absorption loss, such as GSST, GSSe, Sb_2Se_3 and Sb_2S_3. We note that among current PCMs, Sb_2S_3 shows the widest bandgap, hence the lowest operational wavelength. While very large number of levels (up to 64) have been demonstrated using electrical control, none of the research papers tested multi-level repeatability except for[23], where huge variations were reported. Our work is the first work that uses electrical control and shows a consistent and reliable multi-level operation up to 32 levels.

Supplementary Table 4: PCM integrated photonic device performance comparison

Ref	Year	PCM	Structure	ER (dB)	IL (dB)	Energy per switch (nJ)	PCM length (μm)	Optical BW (nm)	Number of levels (repeat-ability study)	Number of cycles
**Optically actuated phase transition**										
30	2015	GST	MRR 1 × 1	10	N.R.	0.533 (3.1) [c]	1.0	< 1	8 (no)	50
31	2017	GST	MRR 1 × 1	> 5.0	5.1 / 4.3 [a]	0.19 (17.1) [c]	60.0	< 1	7 (no)	1,000
32	2018	GST	WG 1 × 1	1.5	N.R.	0.68	2	N.R.	34 (yes)	10
33	2018	GST	MRR 1 × 1	33	0.54	0.62 (0.2) [c]	2	< 1	6 (no)	> 2
10	2019	GST	MRR 1 × 2	14	0.75 / 0.46 [a]	0.25 (11) [c]	25.0	< 1	21 (no)	> 2
34	2019	GST	WG 1 × 1	9.0	1.5	0.7	3.6	> 6 [b]	10 (no)	N.R.
35	2019	GSST	MRR 1 × 1	42	< 0.5	N.R.	5 [b]	< 1 [b]	2 (no)	> 3
36	2021	GST	MZI 2 × 2	8.0	0.5	14 (9.5×10^5) [c]	43.0	N. R.	N.R.	600
11,12	2021	GST	WG 1 × 1	16	0.9	0.38	10.0	> 40 [b]	88 (yes)	1,000
37	2021	GST	Crossing	N.R.	0.4	0.8	2	N.R.	400 (no)	N.R.
**Electrically actuated phase transition**										
38	2017	GST	WG 1 × 1	1.2	4.8	20 (7.2×10^6) [c]	1	> 100	2 (no)	~10
39,40	2019	GST	MMI 1 × 1	10.4	1	10 (9) [c]	1	> 100	5 (no)	> 1500
41	2020	GST	MRR [f] 1 × 1	14.7	0.06	8 (78) [c]	3	< 0.1	2 (no)	> 500

24	2021	Sb_2Se_3	MZI [f]<br>2 × 2	6.5/<br>15.0 [a]	> 0.3	176<br>(3.8 × 10^3) [c]	> 100 [b]	> 15 [b]	9 (no)	> 125
42	2022	GST	MMI<br>1 × 1	16.9	3.7	4.5<br>(4.08) [c]	1	< 5 [b]	37 (no)	N.R.
6	2022	GST	DC [f]<br>2 × 2	10.0	2.0	380<br>(6.8 × 10^3) [c]	50	> 30	2 (no)	> 2,800
23	2022	Sb_2Se_3	MRR [f]<br>1 × 1	14.0	0.33	9.25<br>(1.4 × 10^3) [c]	6	< 0.1	14 (yes, large level variation)	> 1,100
43	2022	GSSe	WG<br>1 × 1	12	0.12	400	80	> 100 [b]	16<br>(unclear)	5 × 10^5
44	2023	GST	MZI [f]<br>2 × 2	20	1	81<br>(1.9× 10^3) [c]	10	> 50 [b]	64 (no)	> 3
**This work**	2023	Sb_2S_3	MZI, MRR, DC	> 10	< 1	56<br>(3.4 × 10^6<br>1.7× 10^3) [c, e]	79	> 40	32 (yes)	> 800

(ER: extinction ratio, IL: insertion loss, BW: bandwidth, MMI: Multi-mode interferometer, MZI: Mach-Zehnder Interferometer, MR(D)R: Micro-ring(disk) resonator, WG: waveguide, DC: directional coupler, N.R.: not reported)

Note: here, only devices with reversible switching are compared. Footprint refers to the total device length. [a]For drop and through ports, respectively. [b]Estimated from figures. [c]Energy per switching event for amorphization (crystallization). [d]The study did not perform multiple runs to verify the multilevel reliability. [e]For ring resonators. A smaller crystallization energy was achieved with shorter 100-μs crystallization pulses. [f]This work contains multiple devices; only one device is picked for easy comparison.

We then compare our Sb_2S_3-based phase shifter with the state-of-the-art using other tuning mechanisms (*Supplementary Table 5*), such as thermo-optic effect, free-carrier effect, electro-optic effect, MEMS, and ferroelectric effect. We note that the modulation bandwidth/rising time are also important metrics in the traditional optical communication application. However, we would like to emphasize that PCMs will never be used as a high-speed modulator, rather should be viewed as a semi-static programmable component. Therefore, those metrics are not compared. *Supplementary Table 5* below reveals that PCMs are an excellent candidate for semi-static application in terms of the footprint, insertion loss, extinction ratio, and energy consumption. Intuitively, the energy benefit comes from their zero static power due to the non-volatile nature. We further note that MEMS also have very promising power budgets because it is electrostatic, but it is generally difficult to fabricate due to their suspended nature.

Supplementary Table 5: Compare our Sb_2S_3-based phase shifter with other tuning mechnisms

Ref	Year	Structure	Mechanism	ER (dB)	IL (dB)	Energy per second (nJ/s)	L_π (μm)	V_π (V)	Nonvolatile
[45]	2013	MZI	TO	> 20	0.5	1.27×10^7	10	11.9	No
[46]	2014	MZI	TO	35	0.23	2.48×10^7	61.6	4.36	No
[47]	2019	MZI	TO	35	< 0.4	2.28×10^7	100	4.77	No
[48]	2020	MDR	TO	7.0	0.8	1.5×10^7	12.8	N.R. ^{c}	No
[49]	2005	MZI	FC	4.5	~10	2.7×10^9	1.3×10^4	3.3	No
[50]	2005	MRR	FC	15	< 0.1	2.5×10^5	< 75	0.94 ^{c}	No
[51]	2014	MRR	FC	14 ^{b}	~ 0	9.7×10^6	35	5 ^{c}	No
[52]	2018	MZI	EO polymer	> 25	8.2	8.5×10^6	1.5×10^4	0.21	No
[53]	2019	MRR	MEMS+ Plamonics	> 20	2.0 (0.1) ^{a}	600	4	1	No
[54]	2021	MZI	MEMS	35 ^{b}	0.33	1	50	10.7	No
[55]	2018	MZI	EO LN	30	< 1	2.59×10^7	2.0×10^4	1.4	No
[56]	2019	MZI	EO LN/Si	40	2.5	1.9×10^7	5.0×10^4	7.4	No
[57]	2022	MRR	FE	12 ^{b}	0.07	2.67×10^{-2}	1×10^3	10	Yes, but requires a constant DC bias
**This work**	2023	MZI, MRR, DC	PCM	> 10	< 1	56 (3.4×10^6 1.7×10^3) $^{c, e}$	40	10	Yes

(ER: extinction ratio, IL: insertion loss, BW: bandwidth, MZI: Mach-Zehnder Interferometer, MR(D)R: Micro-ring(disk) resonator, DC: directional coupler, TO: thermo-optic effect, FC: free-carrier effect, EO: electro-optic effect, LN: $LiNbO_3$, FE: ferroelectric effect, N.R.: not reported)

Notes: For devices based on non-volatile effects, energy consumption is estimated as the energy per switching event, assuming one second per switching event. ^{a} For drop and through ports, respectively. ^{b} Energy per switching event for amorphization (crystallization). ^{c}Micro-ring resonators do not require π phase shift to get full modulation, so the number here means voltage to get full modulation.

Section 16. System scalability analysis: physical scale, optical metrics, and thermal crosstalk

We envision to build large-scale PIC systems using our nonvolatile electrically controlled multi-level components. Such systems have the potential to transform the current landscape of various emerging applications, such as optical programmable gate array, optical information processing, and optical interconnect[22]. In this section, we analyze the requirements in terms of the physical scale, optical performance, and thermal crosstalk, and shed light on future development. We focus on a "Reck" type architecture[58,59], a standard architecture for realizing arbitrary unitary transformation in optics. An $N \times N$ "Reck" system is composed with $\frac{N\cdot(N-1)}{2}$ building blocks, each consisting of a tunable beam splitter (usually implemented with an MZI) and a phase shifter for both amplitude and phase control. Other interesting architectures include Benes type[35] architecture for non-blocking switch array and "Clements"[60] type architecture. In the following discussion, we mainly discuss 3D integration technique for photonic-electronic integration, which supports both the highest integration density via flip-chip bonding and the newest CMOS nodes. We note that there are also other integration techniques, such as 2D[61,62], 2.5D[63,64] and monolithic integration[65,66]. The final choice of the architecture depends on the desired application.

(a). Physical scale

The pad size, metal wire routing and individual PIC component footprint are the most important factors limiting the physical scale of the system. Here we discuss all three factors and analyze what is the biggest scale in a 1 cm × 1 cm PIC chip. First, although a relatively large metal pads were used in our experiments (~150 µm), a much smaller pad pitch of 50 µm is easily accessible nowadays in foundries and even down to below 10 µm pitch [67]. Second, an easier metal wire routing requires less control signals and more metal layers. The total number of controls could be reduced from O(N^2) in the individual control scheme to O(N) using a column-row addressing architecture[68]. Moreover, many foundries support multiple metal layers, such as AIM photonics, global foundry and TSMC, enabling such an architecture. Third, our Sb_2S_3-based tunable beam splitter (phase shifter) is 79 µm (40 µm) in length, which could be further reduced to ~34 µm (~18 µm) using a 50-nm-thick Sb_2S_3. It is generally enough to separate devices in the lateral direction by > 10 µm to avoid the optical crosstalk. Therefore, each building block takes 120 µm × 10 µm, leading to a total of 83 × 1000 building blocks. We relax it to 60 × 60 to allow more spaces between the devices and wiring the PIC devices. Considering each building block consists of 2 components, even a 60 × 60 system is already similar to the state-of-the-art

systems[69], where 128 × 128 MEMS elements were used. The column-row addressing architecture leads to ~ $2 \times 60 = 120$ metal pads, which can be rearranged into a 11 × 11 array, occupying only 0.5mm × 0.5 mm chip space. Therefore, we conclude that the system could be further scaled if more compact PIC devices are made and the metal pad size is not the ultimate limitation, but even our current devices are enough to support a 60 × 60 system close to the state-of-the-art.

(b). Optical metrics

The insertion loss α and extinction ratio η (both in dB) requirements are analyzed in this section. In the worst case, the total insertion loss is $\frac{N(N-1)\times\alpha}{2}$ for an $N \times N$ system. Assuming an acceptable system loss of 10 dB, individual building block loss has to be lower than $\alpha = \frac{20}{N(N-1)} = 0.0056\ dB$ for N=60. The non-ideal extinction ratio leads to an optical crosstalk. In the worst case, all the crosstalk constructively interferes with each other, resulting in a crosstalk of $10^{-\frac{\eta}{10}}N$. We assume a crosstalk of less than 0.01 is allowed, and $\eta = -10\text{lg}\frac{0.01}{N} = 37.8dB$. We, however, note that this is an overestimate of the required extinction ratio. Some work has shown that adding additional imperfect components into the PIC circuit can significantly reduce the finite optical crosstalk[70–72]. We conclude that to construct a 60 × 60 system, better optical performance is required: insertion loss < 0.0056 dB and extinction ratio > 37.8dB. The latter could be relaxed at the cost of redundant components. Our programmable unit can reach the performance in theory, but the experimental performance is worse. Reaching these metrics experimentally will constitute the future research direction.

(c) Thermal crosstalks

In this section, we point out that thermal crosstalk is not a problem in PCM systems[73], unlike traditional thermally controlled photonics. PCMs are switched only when the phase transition threshold is reached (~300°C and 525°C for crystallization and amorphization). In our case, localized heat is transferred through the doped silicon heater and will not provide enough heat for nearby components. In the thermal simulation in Supplementary Section 6, heat is clearly only on the two waveguides. At around 1μm from the waveguides, the temperature already drops to only ~200° Celsius, which will not be able to trigger both amorphization and crystallization. In a system, the photonic devices are usually well separated by at least 5~10 μm to eliminate the optical crosstalk, which precludes any thermal residual from neighboring devices. Furthermore, to guarantee no undesired phase transition happens, one can first set all the devices to the crystalline phase, and amorphize the relevant devices. This utilizes the high

temperature, fast cooling requirement of amorphization, which can only be fulfilled when PCMs are close to the heat source. Importantly, the devices operating in the semi-static regime allows enough thermal relaxation time before the next switching so that the heat could hardly build up. We therefore conclude that the PCM PIC system is inherently immune to thermal crosstalk.

Supplementary References

1. Fang, Z. *et al.* Non-Volatile Reconfigurable Integrated Photonics Enabled by Broadband Low-Loss Phase Change Material. *Advanced Optical Materials* **9**, 2002049 (2021).
2. Parize, R. *et al.* In situ analysis of the crystallization process of Sb2S3 thin films by Raman scattering and X-ray diffraction. *Materials & Design* **121**, 1–10 (2017).
3. Constantin-Popescu, C. *et al.* New phase change materials for active photonics. in *Active Photonic Platforms 2022* vol. 12196 26–37 (SPIE, 2022).
4. Teo, T. Y. *et al.* Comparison and analysis of phase change materials-based reconfigurable silicon photonic directional couplers. *Optical Materials Express* **12**, 606–621 (2022).
5. Xu, P., Zheng, J., Doylend, J. K. & Majumdar, A. Low-Loss and Broadband Nonvolatile Phase-Change Directional Coupler Switches. *ACS Photonics* **6**, 553–557 (2019).
6. Chen, R. *et al.* Broadband Nonvolatile Electrically Controlled Programmable Units in Silicon Photonics. *ACS Photonics* **9**, 2142–2150 (2022).
7. Dong, W. *et al.* Wide Bandgap Phase Change Material Tuned Visible Photonics. *Advanced Functional Materials* **29**, 1806181 (2019).
8. Rayleigh, Lord. On the Stability, or Instability, of certain Fluid Motions. *Proceedings of the London Mathematical Society* **s1-11**, 57–72 (1879).
9. Lian, J., Wang, L., Sun, X., Yu, Q. & Ewing, R. C. Patterning Metallic Nanostructures by Ion-Beam-Induced Dewetting and Rayleigh Instability. *Nano Letters* **6**, 1047–1052 (2006).
10. Wu, C. *et al.* Low-Loss Integrated Photonic Switch Using Subwavelength Patterned Phase Change Material. *ACS Photonics* **6**, 87–92 (2019).
11. Wu, C. *et al.* Programmable phase-change metasurfaces on waveguides for multimode photonic convolutional neural network. *Nature Communications* **12**, 96 (2021).
12. Wu, C. *et al.* Harnessing optoelectronic noises in a photonic generative network. *Science Advances* **8**, eabm2956 (2022).

13. Zheng, J., Zhu, S., Xu, P., Dunham, S. & Majumdar, A. Modeling Electrical Switching of Nonvolatile Phase-Change Integrated Nanophotonic Structures with Graphene Heaters. *ACS Applied Materials & Interfaces* **12**, 21827–21836 (2020).
14. Zheng, J. *et al.* Nonvolatile Electrically Reconfigurable Integrated Photonic Switch Enabled by a Silicon PIN Diode Heater. *Advanced Materials* **32**, 2001218 (2020).
15. Erickson, J. R., Shah, V., Wan, Q., Youngblood, N. & Xiong, F. Designing fast and efficient electrically driven phase change photonics using foundry compatible waveguide-integrated microheaters. *Optics Express* **30**, 13673 (2022).
16. Zhang, Y. *et al.* Myths and truths about optical phase change materials: A perspective. *Applied Physics Letters* **118**, 210501 (2021).
17. Gao, K. *et al.* Intermediate Phase-Change States with Improved Cycling Durability of Sb 2 S 3 by Femtosecond Multi-Pulse Laser Irradiation. *Advanced Functional Materials* 2103327 (2021) doi:10.1002/adfm.202103327.
18. Tuma, T., Pantazi, A., Le Gallo, M., Sebastian, A. & Eleftheriou, E. Stochastic phase-change neurons. *Nature Nanotechnology* **11**, 693–699 (2016).
19. Johnson, G. K., Papatheodorou, G. N. & Johnson, C. E. The enthalpies of formation of SbF5(l) and Sb2S3(c) and the high-temperature thermodynamic functions of Sb2S3(c) and Sb2S3(l). *The Journal of Chemical Thermodynamics* **13**, 745–754 (1981).
20. Senkader, S. & Wright, C. D. Models for phase-change of Ge2Sb2Te5 in optical and electrical memory devices. *Journal of Applied Physics* **95**, 504–511 (2004).
21. Njoroge, W. K., Wöltgens, H.-W. & Wuttig, M. Density changes upon crystallization of Ge2Sb2.04Te4.74 films. *Journal of Vacuum Science & Technology A* **20**, 230–233 (2002).
22. Chen, R. *et al.* Opportunities and Challenges for Large-Scale Phase-Change Material Integrated Electro-Photonics. *ACS Photonics* **9**, 3181–3195 (2022).

23. Fang, Z. *et al.* Ultra-low-energy programmable non-volatile silicon photonics based on phase-change materials with graphene heaters. *Nature Nanotechnology* **17,** 842–848 (2022) doi:10.1038/s41565-022-01153-w.

24. Ríos, C. *et al.* Ultra-compact nonvolatile phase shifter based on electrically reprogrammable transparent phase change materials. *PhotoniX* **3**, 26 (2022).

25. Perez, C. *et al.* Dominant Energy Carrier Transitions and Thermal Anisotropy in Epitaxial Iridium Thin Films. *Advanced Functional Materials* **32**, 2207781 (2022).

26. Yang, J., Ziade, E. & Schmidt, A. J. Uncertainty analysis of thermoreflectance measurements. *Review of Scientific Instruments* **87**, 014901 (2016).

27. Liu, J. *et al.* Simultaneous measurement of thermal conductivity and heat capacity of bulk and thin film materials using frequency-dependent transient thermoreflectance method. *Review of Scientific Instruments* **84**, 034902 (2013).

28. Wuttig, M., Bhaskaran, H. & Taubner, T. Phase-change materials for non-volatile photonic applications. *Nature Photonics* **11**, 465–476 (2017).

29. Abdollahramezani, S. *et al.* Tunable nanophotonics enabled by chalcogenide phase-change materials. *Nanophotonics* **9**, 1189–1241 (2020).

30. Ríos, C. *et al.* Integrated all-photonic non-volatile multi-level memory. *Nature Photonics* **9**, 725–732 (2015).

31. Stegmaier, M., Ríos, C., Bhaskaran, H., Wright, C. D. & Pernice, W. H. P. Nonvolatile All-Optical 1 × 2 Switch for Chipscale Photonic Networks. *Advanced Optical Materials* **5**, 1600346 (2017).

32. Li, X. *et al.* Fast and reliable storage using a 5 bit, nonvolatile photonic memory cell. *Optica* **6**, 1-6 (2019).

33. Zheng, J. *et al.* GST-on-silicon hybrid nanophotonic integrated circuits: a non-volatile quasi-continuously reprogrammable platform. *Optical Materials Express* **8**, 1551 (2018).

34. Feldmann, J., Youngblood, N., Wright, C. D., Bhaskaran, H. & Pernice, W. H. P. All-optical spiking neurosynaptic networks with self-learning capabilities. *Nature* **569**, 208–214 (2019).

35. Zhang, Q. *et al.* Broadband nonvolatile photonic switching based on optical phase change materials: beyond the classical figure-of-merit. *Optics Letters* **43**, 94 (2018).

36. Delaney, M. *et al.* Nonvolatile programmable silicon photonics using an ultralow-loss Sb2Se3 phase change material. *Science Advances* **7**, eabg3500 (2021).

37. Feldmann, J. *et al.* Parallel convolutional processing using an integrated photonic tensor core. *Nature* **589**, 52–58 (2021).

38. Kato, K., Kuwahara, M., Kawashima, H., Tsuruoka, T. & Tsuda, H. Current-driven phase-change optical gate switch using indium–tin-oxide heater. *Applied Physics Express* **10**, 072201 (2017).

39. Zhang, H. *et al.* Nonvolatile waveguide transmission tuning with electrically-driven ultra-small GST phase-change material. *Science Bulletin* **64**, 782–789 (2019).

40. Zhang, H. *et al.* Miniature Multilevel Optical Memristive Switch Using Phase Change Material. *ACS Photonics* **6**, 2205–2212 (2019).

41. Zheng, J. *et al.* Nonvolatile Electrically Reconfigurable Integrated Photonic Switch Enabled by a Silicon PIN Diode Heater. *Advanced Materials* **32**, 2001218 (2020).

42. Wu, D. *et al.* Resonant multilevel optical switching with phase change material GST. *Nanophotonics* **11,** 3437-3446 (2022) doi:10.1515/nanoph-2022-0276.

43. Meng, J. *et al.* Electrical Programmable Low-loss high cyclable Nonvolatile Photonic Random-Access Memory. (2022) doi:10.48550/arXiv.2203.13337.

44. Zhang, C. *et al.* Nonvolatile Multilevel Switching of Silicon Photonic Devices with In2O3/GST Segmented Structures. *Advanced Optical Materials* **11**, 2202748 (2023).

45. Watts, M. R. *et al.* Adiabatic thermo-optic Mach–Zehnder switch. *Optics Letters* **38**, 733 (2013).

46. Harris, N. C. *et al.* Efficient, compact and low loss thermo-optic phase shifter in silicon. *Optics Express* **22**, 10487–10493 (2014).

47. Jacques, M. *et al.* Optimization of thermo-optic phase-shifter design and mitigation of thermal crosstalk on the SOI platform. *Opt. Express, OE* **27**, 10456–10471 (2019).

48. Zhang, W. & Yao, J. Photonic integrated field-programmable disk array signal processor. *Nat Commun* **11**, 406 (2020).

49. Liao, L. *et al.* High speed silicon Mach-Zehnder modulator. *Optics Express* **13**, 3129–3135 (2005).

50. Xu, Q., Schmidt, B., Pradhan, S. & Lipson, M. Micrometre-scale silicon electro-optic modulator. *Nature* **435**, 325–327 (2005).

51. Qiu, C., Gao, W., Soref, R., Robinson, J. T. & Xu, Q. Reconfigurable electro-optical directed-logic circuit using carrier-depletion micro-ring resonators. *Optics Letters* **39**, 6767–6770 (2014).

52. Kieninger, C. *et al.* Ultra-high electro-optic activity demonstrated in a silicon-organic hybrid modulator. *Optica* **5**, 739–748 (2018).

53. Haffner, C. *et al.* Nano–opto-electro-mechanical switches operated at CMOS-level voltages. *Science* **366**, 860–864 (2019).

54. Edinger, P. *et al.* Silicon photonic microelectromechanical phase shifters for scalable programmable photonics. *Optics Letters* **46**, 5671 (2021).

55. Wang, C. *et al.* Integrated lithium niobate electro-optic modulators operating at CMOS-compatible voltages. *Nature* **562**, 101–104 (2018).

56. He, M. *et al.* High-performance hybrid silicon and lithium niobate Mach–Zehnder modulators for 100 Gbit s −1 and beyond. *Nature Photonics* **13**, 359–364 (2019).

57. Geler-Kremer, J. *et al.* A ferroelectric multilevel non-volatile photonic phase shifter. *Nature Photonics* **16**, 491–497 (2022).

58. Harris, N. C. *et al.* Linear programmable nanophotonic processors. *Optica* **5**, 1623 (2018).

59. Reck, M., Zeilinger, A., Bernstein, H. J. & Bertani, P. Experimental realization of any discrete unitary operator. *Physical Review Letters* **73**, 58–61 (1994).

60. Clements, W. R., Humphreys, P. C., Metcalf, B. J., Kolthammer, W. S. & Walsmley, I. A. Optimal design for universal multiport interferometers. *Optica* **3**, 1460 (2016).

61. Li, H. *et al.* A 112 Gb/s PAM4 Silicon Photonics Transmitter with Microring Modulator and CMOS Driver. *Journal of Lightwave Technology* **38**, 131–138 (2020).

62. Li, H. *et al.* A 106 Gb/s 2.5 V<inf>ppd</inf> Linear Microring Modulator Driver with Integrated Photocurrent Sensor in 28nm CMOS. in *2022 Optical Fiber Communications Conference and Exhibition (OFC)* 1–3 (2022).

63. Abrams, N. C. *et al.* Silicon Photonic 2.5D Multi-Chip Module Transceiver for High-Performance Data Centers. *Journal of Lightwave Technology* **38**, 3346–3357 (2020).

64. He, H., Xue, H., Sun, Y., Liu, F. & Cao, L. Design and realization of multi-channel and high-bandwidth 2.5D transmitter integrated with silicon photonic MZM. *Journal of Lightwave Technology* **40,** 5201–5215 (2022) doi:10.1109/JLT.2022.3175518.

65. Moazeni, S. *et al.* A 40-Gb/s PAM-4 Transmitter Based on a Ring-Resonator Optical DAC in 45-nm SOI CMOS. *IEEE Journal of Solid-State Circuits* **52**, 3503–3516 (2017).

66. Atabaki, A. H. *et al.* Integrating photonics with silicon nanoelectronics for the next generation of systems on a chip. *Nature* **556**, 349–353 (2018).

67. Zhang, Y., Samanta, A., Shang, K. & Yoo, S. J. B. Scalable 3D Silicon Photonic Electronic Integrated Circuits and Their Applications. *IEEE Journal of Selected Topics in Quantum Electronics* **26**, 8201510 (2020).

68. Ribeiro, A. *et al.* Column-Row Addressing of Thermo-Optic Phase Shifters for Controlling Large Silicon Photonic Circuits. *IEEE Journal of Selected Topics in Quantum Electronics* **26**, 6100708 (2020).
69. Zhang, X., Kwon, K., Henriksson, J., Luo, J. & Wu, M. C. A large-scale microelectromechanical-systems-based silicon photonics LiDAR. *Nature* **603**, 253–258 (2022).
70. Wilkes, C. M. *et al.* 60 dB high-extinction auto-configured Mach–Zehnder interferometer. *Optics Letters* **41**, 5318–5321 (2016).
71. Miller, D. A. B. Perfect optics with imperfect components. *Optica* **2**, 747–750 (2015).
72. Hamerly, R., Bandyopadhyay, S., Sludds, A. & Englund, D. Design of asymptotically perfect linear photonic circuits. in *AI and Optical Data Sciences III* vol. 12019 89–94 (SPIE, 2022).
73. Fang, Z., Chen, R., Zheng, J. & Majumdar, A. Non-Volatile Reconfigurable Silicon Photonics Based on Phase-Change Materials. *IEEE Journal of Selected Topics in Quantum Electronics* **28**, 8200317 (2022).
